# Self‐control strategies to reduce meat consumption: An ecological momentary intervention

**DOI:** 10.1111/aphw.70225

**Published:** 2026-09-25

**Authors:** Alice Elena Seffen, Laura Maria König, Simone Dohle

**Affiliations:** ^1^ Institute for Family Medicine University of Bonn, University Hospital Bonn Bonn Germany; ^2^ Faculty of Psychology University of Vienna Vienna Austria

**Keywords:** ecological momentary intervention, meat, randomized controlled trial, self‐control, self‐regulation, strategy

## Abstract

An increasing number of individuals aim to reduce their meat consumption, but implementing this is challenging. Self‐control strategies could support this change; however, their effectiveness remains understudied. This study examines whether providing self‐control strategies supports motivated individuals in reducing meat intake. Based on the process model of self‐control, we tested whether situational strategies outperform intrapsychic strategies. Additionally, we examined whether combining both strategy types is more effective than either strategy alone. We conducted an ecological momentary intervention including a baseline (Week 1), an intervention (Weeks 2–4), and a follow‐up (Week 9). Participants—156 German students who regularly consumed meat and aimed to reduce their consumption—were randomized to one of four groups: during the intervention, they received either no, intrapsychic, situational, or both strategies. Meat consumption (in grams) was measured through end‐of‐the‐day reports. Two‐part multilevel models showed that receiving strategies (vs. none) and receiving both strategy families (vs. one) increased the odds of meat‐free meals during the intervention, but did not reduce overall meat consumption. All groups meaningfully reduced meat consumption during study participation, even up to the 1‐month follow‐up. Findings challenge the key prediction of the process model and inform the design of future diary interventions.

## INTRODUCTION

Extensive meat consumption in high‐income countries has substantial negative impacts on public health and the environment, posing a serious risk to human well‐being (Godfray et al., 2018). Specifically, high intake of red and processed meat is a risk factor for noncommunicable diseases such as coronary heart disease, type 2 diabetes, and some types of cancer, as well as overall mortality (Clark et al., 2019). In addition, animal‐based foods cause approximately twice the greenhouse gas emissions of plant‐based foods (Xu et al., 2021), and livestock farming significantly contributes to water shortage, deforestation, and biodiversity loss, accelerating climate change (Willett et al., 2019). To meet recommendations for healthy and sustainable diets, high‐income countries need to reduce their meat consumption by more than half (Willett et al., 2019).

Over recent decades, considerable research has studied the motives for eating meat (e.g. Piazza et al., 2015) and reducing meat consumption (e.g. Seffen & Dohle, 2023), showing that factors such as health, environment, and animal welfare concerns, as well as taste, social norms, and cooking skills, play an important role (for reviews see Graça et al., 2019; Stoll‐Kleemann & Schmidt, 2017). Interventions at the individual level have predominantly provided information on the negative health, environmental, or animal welfare consequences (Kwasny et al., 2022). Such information has been effective in debunking meat myths (Seffen, Weingarten, et al., 2026), promoting more favorable attitudes (Carfora et al., 2019), and increasing intentions to reduce meat consumption (e.g. Cordts et al., 2014); however, such information was not necessarily effective in changing real‐world eating behavior (e.g. Weingarten et al., 2022). In the context of meat reduction, a systematic review of Bianchi et al. (2018) concluded that providing information on health or environmental consequences increases motivation but does not change actual meat consumption. A recent meta‐analysis of randomized controlled trials found that only providing health information yields small effects on meat reduction behavior (Di Gennaro et al., 2024). Indeed, theories of (health) behavior change have emphasized that while knowledge is relevant, especially in the preintentional motivational phase, it is typically insufficient when considering the postintentional volitional phase of behavior change (e.g. Heckhausen & Gollwitzer, 1987; Prochaska & DiClemente, 1983; Schwarzer, 2008). Conclusively, focusing solely on information interventions and on increasing motivation alone is a limited approach to changing individuals' meat consumption.

In recent years, awareness of the negative consequences of meat production and consumption has risen (Cleland et al., 2025), and a growing number of individuals report being willing to reduce their meat consumption (Dagevos, 2021; Seffen & Dohle, 2023). Notably, in a recent study, approximately 12% of Germans and Austrians reported the intention to eat less meat, making it the second most frequently mentioned dietary avoidance goal (Ruf & König, 2025). This suggests that a considerable proportion of the population has entered the volitional phase of behavior change. However, forming an intention is rarely sufficient for behavior change. Across domains, people implement only about half of their intentions (Sheeran & Webb, 2016), a phenomenon known as the intention–behavior gap. In the domain of eating behavior, individuals often even fail to realize an intention that they stated only 3 h ago (Aulbach et al., 2024). The intention–behavior gap also applies specifically to the intention to reduce meat consumption (Çoker & van der Linden, 2020; Linder et al., 2026). Research from the United Kingdom (Laffan et al., 2023) and Germany (Seffen, Blase, & Dohle, 2026) suggests that certain contexts particularly challenge the implementation of the intention to eat less meat, such as when eating out, eating with others, having dinner, on Sundays, at special occasions or barbecues, and when meat craving is high. These findings highlight the need for interventions that support motivated individuals in acting consistently with their goals.

### Self‐regulation and self‐control

Self‐regulation is central in the postintentional, volitional phase of behavior change. It is defined as the “dynamic process of determining a desired end state and taking action to move toward it while monitoring progress along the way” (Inzlicht et al., 2020, p. 320) and includes techniques such as goal setting, planning, monitoring, and self‐control.

Two studies investigated the effects of goal setting on reducing meat consumption in samples of young adults. Monitoring a meat‐free pledge for 1 month (Piazza et al., 2022) or participating in a 1‐month meat reduction challenge (Marty et al., 2025) reduced meat consumption during the intervention, but effects faded at the 1‐ and 3‐month follow‐ups, respectively. Regarding planning, two studies found that implementation intentions—if‐then plans such as “If I go to the supermarket to buy lunch, then I will choose a vegetarian salad”—helped committed individuals reduce their meat consumption over a period of 1 week (Rees et al., 2018) and even up to 1 month after the intervention (Loy et al., 2016). Furthermore, a multicomponent intervention combining aspects of goal setting, planning, monitoring, and self‐control was tested against a monitoring‐only control group (Frie et al., 2022). While both groups reduced their meat consumption during the intervention, those receiving the multicomponent intervention showed greater reductions. However, the intervention effect was not maintained 1 month later. In summary, existing studies suggest that self‐regulation interventions can support short‐term reductions in meat consumption. Yet none of the interventions reviewed has examined the role of self‐control in particular.

Self‐control is a part of self‐regulation. While goal setting, planning, and monitoring can occur without conflict, self‐control becomes relevant when a long‐term goal competes with a momentary desire (Inzlicht et al., 2020). For example, a person's long‐term goal of reducing meat consumption may directly compete with a momentary craving triggered by the smell of a meat dish in a restaurant. Qualitative research indicates that motivated meat reducers often encounter such self‐control conflicts in their daily life (Wehbe et al., 2022). Self‐control strategies are means to help individuals prioritize a long‐term goal over short‐term gratification (Inzlicht et al., 2020), and their use is associated with successful goal attainment across domains (Hennecke & Bürgler, 2020; Milyavskaya et al., 2021; Sheeran & Webb, 2016). Thus, self‐control strategies could play a crucial role in supporting individuals who aim to reduce their meat consumption.

### The process model of self‐control

The process model of self‐control (Duckworth et al., 2014; Duckworth, Gendler, & Gross, 2016) distinguishes two broad types of self‐control strategies: situational strategies and intrapsychic strategies. Situational strategies include situation selection (choosing specific situations) and situation modification (changing aspects of the situation). For instance, a person could choose a vegetarian‐friendly restaurant (situation selection) or bring meat substitutes to a barbecue (situation modification) to make goal attainment easier. Intrapsychic strategies include attentional deployment (guiding attention toward/away from certain aspects), cognitive change (reframing how one thinks about a temptation), and response modulation (using willpower). When confronted with tempting meat options at a restaurant, a person may focus on vegetarian dishes (attentional deployment), think of potential health consequences (cognitive change), or, as a last resort, rely on willpower to suppress the desire (response modulation). Situational strategies are usually used *before* a self‐control conflict arises, whereas intrapsychic strategies are typically used once the conflict is present. Because situational strategies intervene earlier in the impulse‐generation cycle and can *prevent* a self‐control conflict from emerging, the model posits that they are more effective than intrapsychic strategies.

Empirical evidence for this prediction is mixed. Some studies support the superiority of situational strategies in academic (Duckworth, White, et al., 2016), financial (Davydenko & Peetz, 2024), and food‐related domains (Lopez et al., 2021). Other studies, including a meta‐analysis in the financial domain (Davydenko et al., 2021) and an ecological momentary assessment of self‐control conflicts in daily life (Milyavskaya et al., 2021), find the two strategy types to be similarly effective. Consistent with this, a recent experimental study on healthy eating trained participants in either situational strategies (changing aspects of the physical environment) or cognitive strategies (thinking about negative health consequences) and found some support for the effectiveness of both strategies but no evidence for the “earlier is better” hypothesis (Lopez et al., 2026).

Research on the use and effectiveness of self‐control strategies suggests shifting the focus away from identifying “the best” strategy toward recognizing that different strategies may be effective in different situations (Fujita et al., 2020; Hennecke & Bürgler, 2020). Thus, having a large toolbox of strategies that can be applied flexibly appears especially relevant and beneficial (Wenzel et al., 2021; Werner et al., 2025). From this perspective, providing individuals with both types of self‐control strategies may be more effective than teaching only one.

### Self‐control strategies to reduce meat consumption

To date, only two studies have specifically examined self‐control strategies in the context of meat reduction. First, Wehbe et al. (2022) conducted a qualitative online study with motivated meat reducers. Participants reported a broad range of strategies they perceived as helpful, including avoiding situations, meal planning, time management, meal substitution, reminder of consequences, rewards, and social support. Second, Seffen, Blase, and Dohle (2026) conducted two quantitative online studies investigating perceived effectiveness and feasibility of different strategies based on the process model of self‐control. Strategies that were evaluated as promising included selecting specific situations that make meat reduction easier, modifying the meal, guiding attention toward goal‐congruent aspects, and thinking about animal welfare. Trying to modify the social environment and setting a self‐punishment for unmet goals were evaluated rather negatively. A limitation of the aforementioned works is the hypothetical assessment of strategy effectiveness in cross‐sectional online studies. The real‐world effectiveness of self‐control strategies for reducing meat consumption thus remains unclear, highlighting the need for empirical field research.

### The present study

The aims of this research are threefold. First, by focusing on the postintentional, volitional phase of behavior change, the study examines whether providing self‐control strategies in everyday life supports reductions in meat consumption compared with no strategy provision, thereby extending prior work that has largely focused on motivation formation. Second, to test predictions derived from the process model of self‐control and address mixed empirical findings, the study investigates whether situational strategies are more effective than intrapsychic strategies when delivered via a smartphone‐based intervention. Third, addressing the lack of experimental evidence on combined strategy approaches, and building on the notion that a broader “toolbox” of strategies may be particularly beneficial (e.g. Werner et al., 2025), this study tests whether offering both strategy types yields greater reductions than offering either alone.

These aims were examined in a preregistered 9‐week longitudinal randomized controlled trial using ecological momentary assessment/intervention (Dohle & Hofmann, 2017; Heron & Smyth, 2010; Shiffman et al., 2008) delivered via a smartphone app, enabling repeated real‐world assessment of meat consumption and delivery of strategies. This method has the advantage of minimizing recall bias and maximizing ecological validity.

We preregistered the following hypotheses:

Providing self‐control strategies (intrapsychic, situational, both) will reduce meat consumption from baseline to (a) intervention and (b) follow‐up *more* than providing no strategies.

Providing situational strategies will reduce meat consumption from baseline to (a) intervention and (b) follow‐up *more* than providing intrapsychic strategies.

Providing both strategy types will reduce meat consumption from baseline to (a) intervention and (b) follow‐up *more* than providing intrapsychic or situational strategies only.

In addition, the present study also explored potential secondary outcomes such as subjective goal progress, diet satisfaction, habit strength, and perceived behavioral control (PBC) for reducing meat consumption and other health behaviors, as prior research indicates that these constructs are relevant (e.g. de Ridder et al., 2020; Hennecke & Bürgler, 2020; Seffen & Dohle, 2023).

## METHOD

The study received ethical approval from the ethics committee of the Medical Faculty at the University of Bonn (2023‐234‐BO). Participants gave informed consent, could withdraw without explanation at any time, and were debriefed at the end of the study. The study was preregistered (https://osf.io/n5xbp/overview), and all data, code, and materials are available on the Open Science Framework (OSF, https://osf.io/9n287/overview). Analyses were conducted in R version 4.5.1 (R Core Team, 2025).

### Participants

Because the expected effect size was unknown, we based our target sample size on comparable studies (Duckworth, White, et al., 2016, ~50/group) and increased this target by 25%, resulting in 63 participants per group and 252 in total. As preregistered, recruitment was conducted with the goal of reaching this target sample size and was terminated either when the target was reached or when a preregistered stopping date for recruitment was met (whichever comes first).

Participants were recruited from German universities via mailing lists and flyers. Recruitment materials stated that we were seeking regular meat eaters who are interested in reducing their meat consumption. We focused on students because reducing meat consumption is a common goal among young adults (Hielkema & Lund, 2021; Richter et al., 2025), and this life stage offers opportunities for adopting new dietary habits (Hartmann et al., 2014; van den Berg et al., 2022). We targeted regular meat eaters to ensure that participants had sufficient room for meaningful reductions and encounter opportunities to apply self‐control strategies in daily life. Lastly, including willing participants was crucial because self‐control strategies are expected to be effective in the postintentional stage. A screening questionnaire (administered via QuestionPro) thus assessed the following inclusion criteria: being a student, ≥18 years old, consuming meat greater than or equal to four times/week, and being willing to reduce meat consumption.

Participants were compensated depending on the number of meat diaries and questionnaires they completed, receiving €0.50 for each entry. Participants who achieved at least 80% compliance received an additional €10 bonus (max. €29).

### Study design and procedure

**FIGURE 1:**
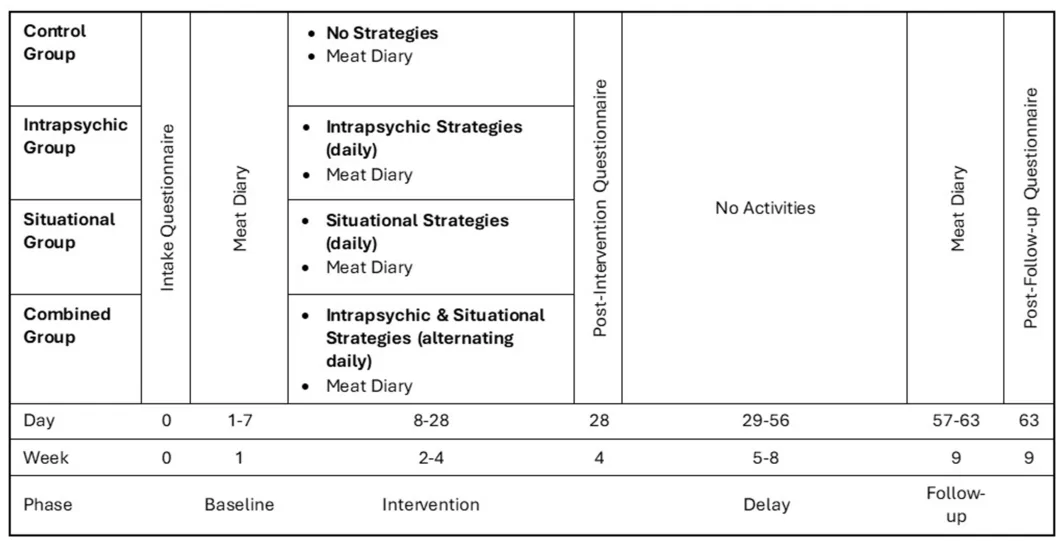
Study design.

We conducted an ecological momentary intervention (Heron & Smyth, 2010), that is, a longitudinal randomized controlled trial, using the smartphone application mPath (Mestdagh et al., 2023). The study included a baseline (Week 1), an intervention (Weeks 2–4), and a follow‐up (Week 9). After baseline, participants were randomized 1:1:1:1 to receive either (1) no strategies, (2) intrapsychic strategies, (3) situational strategies, or (4) both strategy types. Participants completed daily end‐of‐day meat diaries during baseline, intervention, and follow‐up (see Figure 1).

After the screening, eligible participants were instructed to download the app and start with the intake questionnaire (Day 0). This questionnaire assessed demographics, psychological variables, and secondary outcomes. Participants were instructed to eat as usual during baseline to capture their typical meat consumption.

During baseline (Days 1–7), participants completed a daily meat diary at the end of each day. At 7 p.m., participants received a notification prompting them to complete the diary, followed by reminder notifications after 1 and 2 h. The diary could be completed between 7 p.m. and midnight, and participants were instructed to fill it out only after eating dinner. If a diary entry was missed, participants were allowed to enter the information on the following day but not later.

During the intervention (Days 8–28), all participants were instructed to actively reduce meat consumption. Participants in the experimental groups (intrapsychic, situational, and combined) received a daily tip intended to support meat reduction and were instructed to try to apply the strategy. Depending on group assignment, tips included intrapsychic strategies, situational strategies, or both (on alternating days). Participants in the control group did not receive self‐control strategies but a message reminding them to complete the meat diary. The messages (tip or reminder) were sent at 8 a.m., with additional notifications after 1 and 2 h. Messages could be opened multiple times until 7 p.m. Throughout the intervention phase, all participants continued to complete the daily meat diary.

A post‐intervention questionnaire was sent to participants on the evening of Day 28. The questionnaire included a manipulation check and secondary outcomes.

One month after the intervention, participants completed a 1‐week follow‐up assessment of meat consumption (Days 57–63), again using daily meat diaries. No strategies (or reminders) were provided during this phase. On the evening of Day 63, participants were asked to complete a post‐follow‐up questionnaire.

### Measures

#### Dependent variable: Meat consumption

Meat consumption in grams was measured through end‐of‐the‐day diary reports. Meat was defined to include products such as beef, veal, pork, and poultry (e.g. chicken), as well as processed meat products such as cold cuts and sausages but not fish. Participants were first asked whether they had eaten meat for breakfast (*yes*/*no*/*I did not have breakfast*). If they indicated *yes*, they were subsequently asked how many grams of meat they had eaten for breakfast. Grams were indicated using the following categories: less than 50, approx. 100, approx. 150, approx. 200, approx. 250, approx. 300, and more than 350 g. These questions were repeated for lunch, dinner, and snacks. To support participants in estimating meat quantities, we provided a help sheet with examples of common German meat products (based on Haftenberger et al., 2010) and tips for estimating amounts of grams (see OSF).

#### Additional variables

Following the recommendations of de Ridder et al. (2020) for self‐control interventions and the literature on reducing meat consumption (e.g. Seffen, Blase, & Dohle, 2026; Seffen & Dohle, 2023), we assessed several additional variables. We measured motivation, trait self‐control, nutrition app use, and demographics to characterize the sample and to examine baseline equivalence across experimental groups. As secondary outcomes, we assessed subjective outcomes (i.e. subjective goal progress and diet satisfaction) as well as more proximal outcomes (i.e. habit strength and PBC for reducing meat consumption), which could provide insights into the mechanisms underlying intervention effects. We also assessed PBC for other health behaviors to explore potential spill‐over effects (see also Dohle & Hofmann, 2019).

### Intake questionnaire

#### Motivation quantity

Using a single item, participants indicated their motivation to reduce meat consumption on a scale ranging from 1 (*low*) to 7 (*high*).

#### Motivation quality

We assessed reasons for participants' motivation to reduce meat consumption. The items read “I am motivated to reduce my meat consumption for [health/environmental/animal welfare] reasons” (1 = *fully disagree*, 7 = *fully agree*). An additional item assessed whether participants were motivated by other reasons (*yes*/*no*); if so, they could specify these in a free‐text field.

#### Habit strength

We used the four‐item Self‐Reported Habit Index from Gardner et al. (2012) to measure how automatic meat consumption occurred. A sample item reads “Eating meat is something I do automatically” (1 = *fully disagree*, 7 = *fully agree, McDonald's ωwithin* = .87, *ωbetween* = .98).

#### Trait self‐control

We used the German adaptation of the 13‐item short form of the Self‐Control Scale (SCS‐K‐D) to measure trait self‐control (Bertrams & Dickhäuser, 2009; Tangney et al., 2004). A sample item reads “I am good at resisting temptation” (1 = *not at all*, 5 = *very much, ω* = .82).

#### Diet satisfaction

Diet satisfaction was assessed with a single item: “How satisfied are you with your current diet?” (1 = *unsatisfied*, 7 = *very satisfied*).

#### PBC

PBC regarding meat reduction was measured with a single item: “I believe that I am able to reduce my meat consumption” (1 = *fully disagree*, 7 = *fully agree*).

#### Nutrition app use

Participants reported their prior experience of using nutrition apps using a single‐item measure of the stages of behavioral adoption (see König et al., 2018). This measure was included to characterize participants' familiarity with digital food‐monitoring tools.

#### Demographics

We assessed gender, age, height, weight, income, study subject, semester and intended degree, household size, and responsibility for grocery shopping (gatekeeper status).

### Post‐intervention questionnaire

#### Manipulation check

After the intervention phase, participants in the experimental groups (i.e. excluding the control group) answered two separate items: “How often have you been presented with strategies over the past three weeks that can be used [before/during] a challenging eating situation?” Both items were rated on a 7‐point Likert scale (1 = *never*, 7 = *always*).

#### Strategy evaluation

Participants in the experimental groups indicated overall perceived helpfulness of receiving the strategies (1 = *not helpful*, 7 = *very helpful*). In addition, they indicated how effective (1 = *not effective*, 7 = *very effective*) and feasible (1 = *very difficult*, 7 = *very easy*) they found the strategies and to what extent they intended to use the strategies in the future (1 = *not at all*, 7 = *absolutely*).

#### Subjective goal progress

We measured subjective goal progress with three items (taken from Koestner et al., 2012; Werner et al., 2016), e.g. “I have made a lot of progress toward this goal” (1 = *fully disagree*, 7 = *fully agree, ωwithin* = .82, *ωbetween* = .93).

#### PBC for other goals

Using four items, participants indicated to what extent they believed they were able to eat fewer sweets, eat more whole grains, reduce sedentary behavior, and increase physical activity (1 = *fully disagree*, 7 = *fully agree*).

In addition, the post‐intervention questionnaire reassessed habit strength, diet satisfaction, and PBC regarding meat reduction using the same measures as in the intake questionnaire.

### Post‐follow‐up questionnaire

The post‐follow‐up questionnaire reassessed habit strength, diet satisfaction, PBC regarding meat reduction and other health behaviors, and subjective goal progress. All measures corresponded to those used in the intake and post‐intervention questionnaires.

### Intervention material

The self‐control strategies were based on the process model of self‐control and the subcategories identified by Seffen, Blase, and Dohle (2026), who applied the model to strategies for reducing meat consumption. Both strategy types, intrapsychic and situational, were represented by five strategy categories each. Situational strategies included situation selection and situation modification. Situation selection comprised (1) approaching specific situations and (2) avoiding specific situations. Situation modification included (3) modifying the meal, (4) modifying the social environment, and (5) modifying the somatic environment. Intrapsychic strategies covered attentional deployment, cognitive change, and response modulation. Attentional deployment included (1) focusing on goal‐congruent aspects and (2) ignoring goal‐incongruent aspects. Cognitive change comprised thinking about (3) the positive consequences of reducing meat consumption and (4) the negative consequences of eating meat. Response modulation was represented by (5) using willpower.

Each message consisted of a strategy description and a concrete application example (“tactic,” Werner & Ford, 2023). For example, “Tip of the day: Seek out situations that make it easier for you to avoid meat. Example: Choose restaurants that offer a wide range of appealing vegetarian dishes” (approaching specific situations). For each of the five strategy categories, we created four application examples, resulting in 20 messages per strategy type (see Table S1).

The intervention phase lasted 21 days. Participants in the intrapsychic and situational groups received one daily tip drawn randomly without replacement from the respective pool of 20 messages; on Day 21, one randomly selected tip was repeated. Participants in the combined group received tips on alternating days, with one randomly drawn intrapsychic or situational tip per day. On Day 21, it was randomly determined whether an intrapsychic or situational tip was shown.

### Data analysis

Multi‐item scales (trait self‐control, habit strength, and subjective goal progress) were averaged. The BMI was calculated using the common formula of weight/(height)^2^. As a randomization check, we compared the four groups regarding demographics and psychological variables at baseline. We calculated analyses of variance (ANOVAs) for continuous variables and a chi‐square tests for categorical variables. For the manipulation check, we expected strategy exposure ratings to match group assignment: Situational and combined groups should report greater exposure to *before* strategies than the intrapsychic group, whereas intrapsychic and combined groups should report greater exposure to *during* strategies than the situational group. We tested this with two one‐way ANOVAs with Bonferroni‐corrected post hoc tests. In addition to the preregistered analysis, we computed a mixed ANOVA (Group × Rating: before vs. during) with Bonferroni‐corrected post hoc tests.

Due to the nested data structure where meat consumption (Level 1) is repeatedly measured in individuals (Level 2), we used multilevel models to test our hypotheses. We computed two models, one comparing baseline and intervention ([Link], [Link]) and one comparing baseline and follow‐up ([Link], [Link]). We deviate from the preregistered linear multilevel models and instead applied two‐part multilevel models that account for the semi‐continuous (i.e. zero‐inflated and right‐skewed) outcome (see Ruf et al., 2021). The two‐part multilevel model is a novel approach that allows for studying eating behavior as a dual process, distinguishing between (a) a “zero part”: a multilevel logistic regression predicting whether meat is eaten (0 or more than 0 g) and (b) a “continuous part”: a multilevel gamma regression predicting how much meat is eaten if meat is eaten. In contrast to the typical logistic regression, the zero part of this model predicts the probability of *not* eating meat. We used the R package brms (Bürkner, 2017), which uses Bayesian inference, and applied the default priors.

We entered meat intake in grams consumed at main meals (breakfast, lunch, and dinner) as the outcome variable, phase (0 = baseline and 1 = intervention or 1 = follow‐up) as Level 1 predictor, group contrasts (using Helmert coding: C1: strategies vs. control; C2: situational vs. intrapsychic; C3: combined vs. single) as Level 2 predictors, and cross‐level interaction terms (phase * group contrast). To test our hypotheses, the interaction terms are crucial as they represent group differences in changes in (a) meat‐free meals and (b) meat portion size.

Following Ruf et al. (2021), we first checked the convergence of the multilevel two‐part models through visual inspection of density and trace plots and through the Rhat parameter. Random intercept and random slope models were compared using leave‐one‐out cross‐validation (Vehtari et al., 2017). A predictor is interpreted as meaningful if the 95% credible interval does not include zero.

Regarding secondary outcomes, we conducted linear multilevel models for repeatedly measured outcomes, that is, diet satisfaction, habit strength, and PBC of reducing meat consumption, entering phase as Level 1 predictor, group contrasts as Level 2 predictors, and their interaction terms. For outcomes not measured at baseline, that is, subjective goal progress and PBC for other health behaviors, we computed linear regression models entering group contrasts as predictors. For all frequentist‐based analyses, we applied the standard significance criterion of *p* < .05.

## RESULTS

### Participants

**FIGURE 2:**
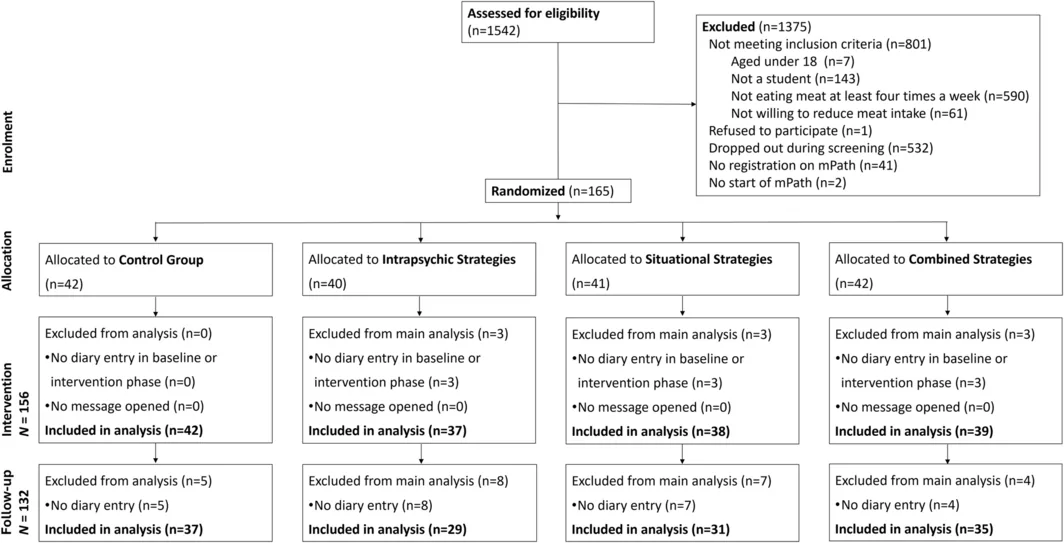
CONSORT flow diagram.

In accordance with the preregistration, recruitment was terminated on the predefined end date (December 31, 2024), at which point the target sample size had not been reached. Of 1542 screening starts, 165 participants met inclusion criteria and began the study, see Consolidated Standards of Reporting Trials (CONSORT) flow diagram in Figure 2. For analysis, we excluded participants who did not complete at least one baseline diary and one intervention diary (*n* = 9). In addition, we checked whether at least one message had been opened during the intervention phase; this was true for all participants. Thus, the final sample consisted of 156 individuals. Mean age was 24.61 (*SD* = 6.28), and 51.3% of the participants identified as women, 47.4% as men, and 1.3% as non‐binary. Participants had a mean BMI of 24.28 (*SD* = 4.86), which falls within the normal‐weight range according to World Health Organization classifications (James, 2008). They were motivated to reduce their meat consumption (*M* = 4.99, *SD* = 1.07) and showed a medium level of trait self‐control (*M* = 3.10, *SD* = 0.60). See Table 1 for more detailed sample characteristics. For baseline–follow‐up analyses, participants with no follow‐up diary entries were excluded (*n* = 24), resulting in 132 participants. Among all participants, 42.3% had never considered using a nutrition app, 5.8% planned to use one in the future, 7.1% had decided against using one, 5.8% were currently using a nutrition app, and 39.1% had used one in the past. Thus, approximately 45% of our sample had experience with digitally recording their food intake.

**TABLE 1:** Baseline characteristics of participants in total and by intervention group.

Characteristic	Total *N* = 156	Control *N* = 42	Intrapsychic *N* = 37	Situational *N* = 38	Combined *N* = 39	Difference test	*p*‐value
Age in years, mean (SD)	24.61 (6.28)	24.3 (5.21)	23.7 (3.94)	24.8 (6.94)	25.6 (8.25)	*F*(3, 152) = 0.61	.608
Income in EUR, mean (SD)	840 (510)	845 (417)	926 (603)	803 (555)	788 (464)	*F*(3, 152) = 0.55	.651
BMI, mean (SD)	24.28 (4.86)	24.3 (4.93)	24.0 (4.70)	24.1 (4.37)	24.7 (5.50)	*F*(3, 152) = 0.14	.936
Household size, mean (SD)	2.54 (1.35)	2.57 (1.38)	2.57 (1.28)	2.79 (1.49)	2.26 (1.23)	*F*(3, 152) = 1.02	.387
Semester, mean (SD)	5.22 (3.79)	5.5 (3.60)	5.62 (4.52)	5.32 (3.82)	4.46 (3.19)	*F*(3, 152) = 0.74	.530
Gender,^a^ *n* (%)						*χ* ^2^(3) = 0.84	.840
Women	80 (51.3)	23 (54.8)	19 (51.4)	21(55.3)	17 (43.6)		
Men	74 (47.4)	19 (45.2)	18 (48.6)	17 (44.7)	20 (51.3)		
Non‐binary	2 (1.3)	0	0	0	2 (5.1)		
Shopping responsibility, *n* (%)						*χ* ^2^(6) = 2.50	.869
Mainly	61 (39.1)	15 (35.7)	13 (35.1)	15 (39.5)	18 (46.2)		
Partly	80 (51.3)	24 (57.1)	19 (51.4)	20 (52.6)	17 (43.6)		
Not	15 (9.6)	3 (7.1)	5 (13.5)	3 (7.9)	4 (10.3)		
Trait self‐control (range: 1–5), mean (SD)	3.10 (0.60)	3.10 (0.58)	3.13 (0.69)	3.11 (0.56)	3.05 (0.58)	*F*(3, 152) = 0.11	.953
Motivation to eat less meat (range: 1–7), mean (SD)	4.99 (1.07)	4.93 (1.09)	4.86 (1.08)	4.89 (1.13)	5.26 (0.94)	*F*(3, 152) = 1.13	.338
Motivation: Health (range: 1–7), mean (SD)	4.12 (1.93)	4.57 (1.98)	4.22 (1.92)	4.05 (2.03)	3.62 (1.73)	*F*(3, 152) = 1.73	.164
Motivation: Environment (range: 1–7), mean (SD)	5.03 (1.39)	4.88 (1.53)	4.95 (1.35)	5.37 (1.13)	4.92 (1.49)	*F*(3, 152) = 1.03	.380
Motivation: Animal welfare (range: 1–7), mean (SD)	5.05 (1.66)	4.86 (1.80)	5.11 (1.61)	4.95 (1.72)	5.31 (1.49)	*F*(3, 152) = 0.56	.640
Habit strength of eating meat (range: 1–7), mean (SD)	4.26 (1.39)	4.10 (1.59)	3.94 (1.32)	4.39 (1.34)	4.62 (1.19)	*F*(3, 152) = 1.87	.137
PBC of reducing meat consumption (range: 1–7), mean (SD)	5.66 (1.04)	5.57 (1.13)	5.81 (0.85)	5.63 (1.28)	5.64 (0.87)	*F*(3, 152) = 0.37	.777
Diet satisfaction (range: 1–7), mean (SD)	3.97 (1.20)	3.86 (1.14)	3.95 (1.13)	4.26 (1.27)	3.85 (1.25)	*F*(3, 152) = 1.03	.381

^a^ For the chi‐square test of gender, two participants who identified as non‐binary were excluded.

### Randomization check, compliance, and manipulation check

The four groups did not differ in sample characteristics, including age, gender, income, BMI, household size, semester, and shopping responsibility (all *p*s ≥ .387). Also, groups did not differ in psychological variables at baseline, including trait self‐control, motivation to eat less meat, habit strength of eating meat, PBC of reducing meat consumption, and diet satisfaction (all *p*s ≥ .137). Results indicate successful randomization (see Table 1 for details).

In total, we analyzed 4826 meat diaries, including 14,478 main meals. Considering the baseline, intervention, and follow‐up phase, participants on average completed 30.94 diaries (*SD* = 5.40, min. = 10, max. = 35), that is, a mean compliance of 88% (*SD* = 15). An ANOVA showed that the four groups did not differ in meat diary compliance, *F*(3, 152) = 2.14, *p* = .098. Participants, on average, opened 19.40 messages (*SD* = 2.97, min. = 7, max. = 21), corresponding to a mean compliance of 92% (*SD* = 14). An ANOVA showed that the four groups differed in compliance with opened messages, *F*(3, 152) = 4.01, *p* = .009, with the situational group showing lower mean compliance than the intrapsychic group (*p* = .015) and the control group (*p* = .025).

The manipulation check (one‐way ANOVAs) revealed that the three experimental groups differed significantly in reported received strategies that can be used *during* a tempting situation, *F*(2, 104) = 8.99, *p* < .001. The intrapsychic group reported higher frequencies than the situational (*p* < .001) and the combined group (*p* = .001). However, groups did not differ in reported strategies that can be used *before* a tempting situation, *F*(2, 104) = 1.50, *p* = .227. The additional mixed ANOVA, which offered greater statistical power, revealed a significant interaction between groups and rating, *F*(2, 104) = 10.37, *p* < .001. As expected, the situational group recognized significantly more *before* strategies than *during* strategies (*M*_diff_ = 0.94, *p* < .001) and the intrapsychic group significantly more *during* than *before* strategies (*M*_diff_ = −0.69, *p* = .042). However, the combined group reported significantly more *before* than *during* strategies (*M*_diff_ = 0.50, *p* = .031), although no significant difference was expected.

Across the three experimental groups, the received strategies were considered moderately helpful (*M* = 3.55, *SD* = 1.48). Groups differed significantly in this evaluation, *F*(2, 104) = 7.384, *p* = .001, with the situational group (*M* = 4.18, *SD* = 1.70) indicating that the strategies were more helpful than the intrapsychic group (*M* = 2.89, *SD* = 1.37, *p* < .001). Detailed results on perceived effectiveness, feasibility, and intention to use the strategies in the future can be found in Figures S1–S3.

### Main analyses

#### Short‐term effects

To investigate short‐term effects, we compared meat consumption at baseline with the intervention phase. The random slope model was preferred over the random intercept model. Results of the Bayesian multilevel two‐part model are shown in Table 2.

**TABLE 2:** Bayesian multilevel two‐part model predicting meat consumption from baseline to intervention.

	Zero part				Continuous part			
	Estimate	*SE*	95% CI		Estimate	*SE*	95% CI	
			*LL*	*UL*			*LL*	*UL*
Fixed effects								
Intercept	**0.38**	**0.06**	**0.27**	**0.50**	**4.72**	**0.03**	**4.67**	**4.78**
Phase (0 = baseline, 1 = intervention)	**0.56**	**0.05**	**0.46**	**0.66**	**−0.12**	**0.02**	**−0.16**	**−0.08**
Strategies versus control	−0.06	0.14	−0.34	0.21	−0.11	0.07	−0.24	0.02
Situational versus intrapsychic	0.22	0.17	−0.11	0.55	0.10	0.08	−0.06	0.26
Combined versus single	−0.11	0.14	−0.38	0.18	−0.05	0.07	−0.20	0.08
Strategies versus control * phase	**0.24**	**0.12**	**0.01**	**0.47**	0.07	0.05	−0.02	0.16
Situational versus intrapsychic * phase	0.13	0.15	−0.15	0.42	−0.05	0.06	−0.17	0.06
Combined versus single * phase	**0.33**	**0.13**	**0.07**	**0.57**	0.07	0.05	−0.03	0.17
Random effects								
SD intercept	**0.55**	**0.06**	**0.45**	**0.66**	**0.31**	**0.02**	**0.27**	**0.36**
SD phase	**0.29**	**0.09**	**0.12**	**0.45**	**0.13**	**0.04**	**0.04**	**0.19**

*Note*: The zero part predicts the log‐odds of a meat‐free meal. The continuous part predicts the log amount of meat eaten in grams if a meal included meat. Credible intervals that do not include zero are presented in bold. *N* = 11,946 observations (meals) across 156 participants.

Abbreviations: CI, credible interval; *LL*, lower limit; *UL*, upper limit.

The zero part of the model predicts the log‐odds of a meat‐free meal. The intercept shows that at baseline, across participants, 59% of the main meals were meat free (*plogis*[.38]). Across participants, during the intervention phase, the odds of meat‐free meals were 1.75 times higher than at baseline (exp[0.56] = 1.75), corresponding to an increase from 59% to 72% of meat‐free meals. Group contrasts show that at baseline, the groups did not differ credibly in their probability of consuming meat‐free meals. Supporting H1a, participants in the strategy groups showed a larger increase in the odds of meat‐free meals compared with the control group (exp[0.24] = 1.27). Contrasting H2a, the situational and intrapsychic groups did not differ credibly in their changes over time. Supporting H3a, the combined strategy group showed a larger increase in the odds of meat‐free meals than the single‐strategy groups (exp[0.33] = 1.39). Random effects indicate substantial individual differences in the baseline probability of avoiding meat meals and how this changed over time.

The continuous part predicts the log amount of meat eaten if a meal included meat. The intercept shows that at baseline, participants ate approx. 112 g of meat per main meal when meat was eaten (exp[4.72] = 112.17). During the intervention phase, the amount of meat eaten per meat meal decreased by approx. 11% (exp[−0.12] = 0.89), corresponding to an average of 99 g per meat meal. Results show no credible baseline differences in meat portion size between groups. Contrasting [Link], [Link], interaction terms showed no credible differences between groups in portion size reductions. Random effects again indicated substantial individual differences in baseline meat portion sizes and changes over time. As sensitivity analyses, we added (a) meat diary compliance and (b) message compliance as covariates to the model, and the result pattern did not change.

We additionally computed marginal expected values to quantify overall meat consumption changes (Table 3). Whereas all groups reduced their meat consumption per meal meaningfully from baseline to intervention, the difference‐in‐difference was not meaningful for the strategies versus control group (*M*Δ = −1.29 [−8.50, 5.85]), situational versus intrapsychic group (*M*Δ = −4.40 [−13.0, 4.11]), and combined versus single groups (*M*Δ = −4.33 [−11.9, 2.84]).

**TABLE 3:** Marginal expected meat consumption reduction per meal in grams by group.

Group	Δ Baseline → Intervention (g)	Δ Baseline → Follow‐up (g)
Combined	−20.8 [−26.7, −15.3]	−24.2 [−32.9, −15.6]
Situational	−18.7 [−25.3, −12.6]	−21.1 [−30.7, −11.6]
Intrapsychic	−14.3 [−20.3, −8.8]	−14.5 [−24.5, −4.79]
Control	−16.7 [−23.3, −10.5]	−20.2 [−29.5, −11.4]

*Note*: Values are estimated from the Bayesian multilevel two‐part models. Brackets show 95% credible intervals. Predictions are estimated at the population level (fixed effects only).

#### Long‐term effects

To investigate long‐term effects, we compared meat consumption at baseline with the follow‐up phase. The random slope model was preferred over the random intercept model. Results are shown in Table 4.

**TABLE 4:** Bayesian multilevel two‐part model predicting meat consumption from baseline to follow‐up.

	Zero part				Continuous part			
	Estimate	*SE*	95% CI		Estimate	*SE*	95% CI	
			*LL*	*UL*			*LL*	*UL*
Fixed effects								
Intercept	**0.36**	**0.06**	**0.24**	**0.48**	**4.72**	**0.03**	**4.66**	**4.79**
Phase (0 = baseline, 1 = follow‐up)	**0.69**	**0.09**	**0.52**	**0.87**	**−0.11**	**0.04**	**−0.19**	**−0.04**
Strategies versus control	−0.14	0.14	−0.41	0.13	−0.10	0.08	−0.25	0.05
Situational versus intrapsychic	0.19	0.19	−0.18	0.55	0.10	0.10	−0.09	0.29
Combined versus single	−0.01	0.15	−0.31	0.28	−0.02	0.08	−0.16	0.14
Strategies versus control * phase	0.15	0.21	−0.25	0.57	0.08	0.08	−0.07	0.24
Situational versus intrapsychic * phase	0.19	0.27	−0.33	0.71	−0.09	0.10	−0.28	0.11
Combined versus single * phase	**0.46**	**0.23**	**0.03**	**0.92**	0.08	0.08	−0.08	0.24
Random effects								
SD intercept	**0.56**	**0.06**	**0.44**	**0.68**	**0.33**	**0.03**	**0.28**	**0.38**
SD phase	**0.76**	**0.10**	**0.56**	**0.97**	**0.24**	**0.04**	**0.15**	**0.32**

*Note*: The zero part predicts the log‐odds of a meat‐free meal. The continuous part predicts the log amount of meat eaten in grams if a meal included meat. Credible intervals that do not include zero are presented in bold. *N* = 5235 observations (meals) across 132 participants.

Abbreviations: CI, credible interval; *LL*, lower limit; *UL*, upper limit.

In the zero part of the model, phase is a credible predictor, showing that at follow‐up, the odds of meat‐free meals were 1.99 times higher than at baseline (exp[0.69] = 1.99). Additionally, H3b was supported, showing that the combined strategy group showed a larger increase in the odds of meat‐free meals than the single‐strategy groups (exp[0.46] = 1.58). In the continuous part, only phase is a credible predictor, showing that from baseline to follow‐up, the amount of meat eaten per meat meal decreased by approx. 10% (exp[−0.11] = 0.90). Contrasting [Link], [Link], interaction terms show no credible differences between groups in portion size reductions. Including meat diary compliance or message compliance as a covariate slightly altered the pattern of results; the combined group no longer showed a greater increase in the odds of meat‐free meals than the single‐strategy groups.

In addition, we computed marginal expected values from the model to quantify overall meat consumption changes (Table 3). Whereas all groups reduced their meat consumption per meal meaningfully from baseline to follow‐up, the difference‐in‐difference was not meaningful for the strategies versus control group (*M*Δ = 0.28 [−10.0, 11.4]), situational versus intrapsychic group (*M*Δ = −6.61 [−20.3, 7.15]), and combined versus single groups (*M*Δ = −6.38 [−17.6, 4.29]).^1^

### Secondary outcomes

Exploratory analyses show that, across groups, diet satisfaction increased and meat‐eating habit strength decreased from baseline to intervention and follow‐up (Table 5). In addition, results suggest some advantages of receiving situational compared with intrapsychic strategies; after the intervention, participants in the situational group showed higher subjective goal progress, a stronger increase in PBC of reducing meat consumption, and a stronger PBC for eating more whole grains. After the follow‐up, participants in the situational group still showed higher subjective goal progress and additionally a stronger decrease in habit strength. Finally, the combined group showed a stronger decrease in habit strength compared with the single‐strategy groups.

**TABLE 5:** Secondary outcomes.

Outcome	Intervention			Follow‐up		
	*N*	Main effect phase	Interactions (contrast * phase)	*N*	Main effect phase	Interactions (contrast * phase)
Subjective goal progress (post only)	149	‐	Situational versus intrapsychic: +0.74*	130	‐	Situational versus intrapsychic: +1.05**
Diet satisfaction (repeated)	156	+0.61***	ns	156	+0.74***	ns
Habit strength of eating meat (repeated)	156	−1.01***	ns	156	−1.46***	Situational versus intrapsychic: −1.00** Combined versus single: −0.64*
PBC of reducing meat consumption (repeated)	156	ns	Situational versus intrapsychic: +0.70*	156	ns	ns
PBC of eating less sweets (post only)	149	‐	ns	130	‐	ns
PBC of eating more whole grains (post only)	149	‐	Situational versus intrapsychic: +0.96**	130	‐	ns
PBC of less sedentary behavior (post only)	149	‐	ns	130	‐	ns
PBC of more physical activity (post only)	149	‐	ns	130	‐	ns

*Note*: Linear regression models were computed for post‐only outcomes. Linear multilevel models with random intercepts were computed for repeated outcomes (i.e. measured at baseline). Numbers represent regression weights.

Abbreviations:‐, not tested; ns, not significant; PBC, perceived behavioral control.

* *p* > .05.

** *p* < .01.

*** *p* < .001.

## DISCUSSION

To promote healthier and more sustainable eating patterns, such as a reduction in meat consumption, it is not only important to increase individuals' motivation, but also to support motivated individuals in implementing their goals in everyday life. This preregistered ecological momentary intervention tested whether delivering daily self‐control strategies supports motivated meat eaters in reducing meat consumption, and whether strategy type matters. Across all conditions (no, situational, intrapsychic, and both strategies), participants substantially reduced meat intake from baseline to the intervention phase and maintained these reductions at the 1‐month follow‐up, accompanied by decreased habit strength of eating meat and increased diet satisfaction. Providing strategies produced a specific benefit for *whether* participants ate meat: receiving strategies (vs. none) and receiving both strategy types (vs. one only) increased the odds of meat‐free meals during the intervention. However, strategy provision did not translate into credible between‐group differences in overall consumption reductions (grams). Contrary to the key prediction of the process model, situational strategies did not outperform intrapsychic strategies in the primary outcome. Taken together, the findings suggest that daily strategy prompts can shift meal‐level choices toward meat‐free options but that broader reductions in meat intake may be driven by more general goal engagement and self‐monitoring processes that were present across all groups.

### Meat consumption reduction across groups

Across groups, participants increased the consumption of meat‐free meals and reduced the portion size of meat in meat meals, resulting in an overall meat consumption reduction that was not only present during the 3‐week intervention phase but also at the 1‐month follow‐up. Thus, participation in this study was associated with sustained meat consumption reduction, which is consistent with healthier and more sustainable dietary patterns.

The meaningful meat reduction in the control group can be attributed to several aspects. Participants were motivated to reduce their intake, reported their intention to do so at the beginning of the study (mere‐measurement effect; Morwitz & Fitzsimons, 2004), and were explicitly instructed to reduce consumption after baseline (goal activation). In addition, they may have found and applied effective self‐control strategies on their own. Also, the daily meat diary—used as a repeated behavioral measure—constituted self‐monitoring of meat consumption. Monitoring is a core component of self‐regulation theories (Carver & Scheier, 1982), facilitating behavior change by increasing awareness of current behavior and enabling comparison with personal goals. It is widely applied in health behavior change interventions, with strong empirical support for its effectiveness (Harkin et al., 2016; Michie et al., 2009). The phenomenon that behavioral measurement can itself unintentionally alter behavior is known as measurement reactivity (French & Sutton, 2010; see also Spangenberg et al., 2016) and may be especially relevant in ecological momentary designs (König et al., 2022). If self‐monitoring occurs in a baseline and intervention phase for both intervention and control groups, this carries the risk of overlooking an actual intervention effect (French et al., 2021; König et al., 2022). Accordingly, our active control group represents a conservative benchmark, capturing the effect of self‐control strategies over and above the effect of monitoring. At the same time, these findings highlight self‐monitoring itself as a potent and scalable intervention component for dietary behavior change (Berry et al., 2021; Burke et al., 2011). Future interventions would benefit from disentangling the effect of monitoring from self‐control strategies.

### The effectiveness of self‐control strategies in reducing meat consumption

We hypothesized that receiving self‐control strategies would help individuals to follow their goal of reducing meat consumption (H1). Results partially support this hypothesis, as participants in the experimental groups showed a stronger short‐term increase in meat‐free meals. However, strategy provision did not affect meat portion size. This suggests that strategies may primarily help with binary food choices (meat vs. no meat) rather than downscaling portions once meat is chosen. One possible reason is that our messages emphasized avoiding meat rather than eating smaller portions, with only one example specifically addressing portion sizes. Furthermore, meat is often consumed in fixed quantities (e.g. one sausage or steak), and asking for half a portion may be seen as socially inappropriate or impractical if this would mean that the remaining meat may go to waste. Importantly, we found no evidence that the intervention backfired by increasing meat portion sizes when meat was consumed. Future interventions may benefit from combining strategies that promote meat‐free meal choices with strategies specifically targeting portion size reduction.

Notably, the effect of strategies on choosing meat‐free meals did not translate into differences in total grams of meat reduced. Overall meat consumption reflects both the probability of choosing a meat‐containing meal and the amount of meat consumed when meat is eaten. Thus, relatively small changes in meal choice (with credible intervals close to zero) did not result in detectable differences in overall meat consumption. This questions the practical impact of providing self‐control strategies when implemented as brief, generic smartphone messages. Although correlational studies consistently link self‐control strategy use to goal attainment, experimental evidence for behavioral effects remains mixed. Consistent with our findings, Lopez et al. (2026) found that participants who received training in self‐control strategies in order to eat healthier showed some positive effects in proximal outcomes (e.g. cravings and resistance) but no consistent effects on actual eating behavior. Similarly, Davydenko and Peetz (2024) found no effects of self‐control strategy provision on spending behavior. Moreover, intervention effectiveness likely depends not only on whether strategies are provided but also on how they are taught, practiced, and used in daily life. More intensive, personalized, or context‐sensitive delivery formats (e.g. meeting up in focus groups or 1:1 sessions) may be required to translate theoretical strategy benefits into robust behavioral change.

Relying on the process model of self‐control, we assumed that providing situational strategies would be more effective than providing intrapsychic strategies (H2). The manipulation check indicated that participants differentiated between strategy types, with the situational group reporting more *before* strategies and the intrapsychic group more *during* strategies, suggesting that the intervention content was perceived as intended. Nevertheless, situational strategies did not yield stronger short‐ or long‐term reductions in either meat‐free meals, portion size, or total meat consumption reduction, despite being evaluated as more helpful.

We found no support for the superiority of situational over intrapsychic strategies. This contradicts the key assumption of the process model (Duckworth et al., 2014; Duckworth, Gendler, & Gross, 2016) and empirical evidence supporting it (Duckworth, White, et al., 2016). Notably, its empirical support comes from a study that compared the provision of situation modification strategies with response modulation. The latter has been questioned as a valid strategy because it largely reflects the outcome of successful self‐control (Werner & Ford, 2023). When situation modification was compared with cognitive change (Lopez et al., 2026) or when multiple situational strategies were compared with multiple intrapsychic strategies, as in the present study, no evidence for the “earlier is better” hypothesis was found. Future experimental work testing this hypothesis should therefore carefully consider which strategy categories are compared.

Extending prior research, we tested whether offering both strategy types is more effective than offering only one (H3). In line with this, participants in the combined condition showed a stronger short‐term increase in meat‐free meals. However, no short‐ or longer term effects on portion size were evident. Whereas overall meat reduction was descriptively largest in the combined group, it did not differ significantly from the single‐strategy groups. These findings provide partial support for the idea that a broader repertoire of self‐control strategies may be beneficial, particularly for avoiding meat completely in a meal. This benefit may stem from increased flexibility in responding to situational demands (Fujita et al., 2020; Hennecke & Bürgler, 2020; Wenzel et al., 2021; Werner et al., 2025).

### Secondary outcomes

Beyond changes in meat consumption, exploratory analyses indicated reductions in habit strength and increases in diet satisfaction across groups. The decrease in automaticity is likely attributable to intensive self‐monitoring, which repeatedly directed attention to this behavior. The increase in diet satisfaction suggests that reducing meat intake was accompanied by a positive evaluation of this dietary shift and did not come at the cost of subjective well‐being.

Situational strategies showed advantages in several secondary outcomes, including subjective goal progress, PBC for reducing meat intake, and for eating more whole grains. The latter finding suggests a positive spill‐over effect (see also Dohle & Hofmann, 2019), whereby learning situational strategies may facilitate improvements in related dietary behaviors. However, no spill‐over effects were observed for the PBC of other health behaviors. Future studies could examine whether more intensive strategy training produces broader cross‐domain effects. Why the advantages of situational over intrapsychic strategies did not translate into behavioral effects remains an open question and warrants further investigation.

### Strengths and limitations

This study has several methodological strengths. First, we employed a theory‐driven, preregistered randomized controlled ecological momentary intervention design that closely resembled real‐world dietary app use. Second, the repeated behavioral assessment across baseline, intervention, and follow‐up enabled sensitive detection of within‐person changes in both the short and longer term. Third, we applied a novel two‐part multilevel modeling approach that allowed us to distinguish between intervention effects on the probability of consuming meat‐free meals and on portion size. Finally, the study followed open science practices, including preregistration and publicly available data, code, and materials, enhancing transparency and reproducibility.

Several limitations need to be acknowledged. The sample consisted of university students, a young, highly educated group, which restricts the generalizability of the findings. As discussed above, the strong effects of daily self‐monitoring—implemented across all conditions—may have reduced between‐group differences and masked strategy‐specific effects. Future studies should therefore disentangle monitoring from strategy provision (e.g. by including a no‐monitoring control condition). We applied performance‐contingent monetary compensation, which may have increased participants' engagement with the intervention; future studies should examine whether similar compliance and intervention effects can be achieved without financial incentives (see also Giese & König, 2024). Although message opening was recorded, strategy use was not assessed directly. Future studies may examine the demand for and use of self‐control strategies in more detail. Examining how often meat‐related self‐control conflicts occur, which strategies are used in which situations, and which strategy–situation combinations are most effective would advance theory and practice. Furthermore, the success of self‐control strategies is likely influenced by individuals' social and physical environments, for example, by social norm perceptions and the availability of meat‐free options; investigating such interactions represents an interesting avenue for future research. Additionally, research using just‐in‐time interventions to deliver self‐control strategies close to challenging eating occasions would be of value. Finally, meat consumption was self‐reported, and no other food categories were assessed. Future interventions should assess overall dietary intake to determine whether reduced meat intake is accompanied by the consumption of healthier alternatives. Furthermore, it would be worthwhile to test self‐control strategies that specifically promote a healthy substitution, such as reducing meat consumption by increasing legume consumption.

## CONCLUSION

This study examined the real‐world usefulness of self‐control strategies for reducing meat consumption within a smartphone‐based intervention. While daily self‐monitoring emerged as a powerful driver of sustained behavior change, self‐control strategies provided small additional benefits, primarily by increasing the likelihood of meat‐free meals rather than reducing overall intake. This suggests that the strategies supported specific binary decisions rather than broader consumption reduction. Situational strategies did not outperform intrapsychic strategies in the primary behavioral outcome, challenging the central prediction of the process model of self‐control. In line with the toolbox theorizing, findings suggest that offering a broader range of strategies may be more beneficial than relying on a single‐strategy type.

These results have practical implications for the design of digital dietary interventions. Given the high familiarity with nutrition apps in young and educated populations, careful and evidence‐based app design may have a substantial impact. Daily self‐monitoring appears to be a scalable and effective core component for motivated individuals seeking to reduce meat consumption. When self‐control strategies are included, they should target both the likelihood of choosing meat‐free meals and the reduction of portion sizes, and interventions may benefit from providing a diverse set of empirically evaluated strategies rather than relying on a single type. Finally, the mode of delivery warrants consideration: While brief generic messages may have limited impact, more personalized and interactive formats may enhance effectiveness but must be balanced against scalability and resource constraints. Overall, findings inform both theory and the development of evidence‐based interventions aimed at promoting healthier and more sustainable diets.

## CONFLICT OF INTEREST STATEMENT

The authors declare no conflicts of interest.

## ETHICS STATEMENT

The study received ethical approval from the ethics committee of the Medical Faculty at the University of Bonn (2023‐234‐BO).

## Supporting information


**Table S1:** Intervention Messages (translated).
**Figure S1:** Perceived Strategy Effectiveness.
**Figure S2:** Perceived Strategy Feasibility.
**Figure S3:** Intention to Use the Strategies in the Future.
**Table S2:** Short‐term Effects: Meal Type as Moderator.
**Table S3:** Long‐term Effects: Meal Type as Moderator.
**Table S4:** Short‐term Effects: Trait Self‐control as Moderator.
**Table S5:** Long‐term Effects: Trait Self‐control as Moderator.
**Table S6:** Short‐term Effects: Motivation as Moderator.
**Table S7:** Long‐term Effects: Motivation as Moderator.

## Data Availability

Data and analysis code are publicly available at the Open Science Framework (OSF): https://osf.io/9n287/overview.

## ENDNOTE

^1^
As exploratory analyses, we additionally examined whether meal type (breakfast, lunch, and dinner), trait self‐control and motivation moderate the short‐ and long‐term intervention effects. Because this required estimating three‐way interactions in already complex two‐part multilevel models, and because the study was not powered to detect such higher order interaction effects, these analyses should be interpreted with caution and are reported in Tables S2–S7.
