# Supplementary material for: Self‐control strategies to reduce meat consumption: An ecological momentary intervention
Source: Appl Psychol Health Well Being. 2026 Sep 25;18(5):e70225. doi: 10.1111/aphw.70225 (PMC13614289; doi:10.1111/aphw.70225)
Supplement: Supplementary file 1 [file APHW-18-0-s001.docx]

**Supplemental Material**

Concerning the article:

**Self-Control Strategies to Reduce Meat Consumption: An Ecological Momentary Intervention**

**Content**

[Table S1: *Intervention Messages (translated)*	2](#_Toc234324945)

[Figure S1: *Perceived Strategy Effectiveness*	6](#_Toc234324946)

[Figure S2: *Perceived Strategy Feasibility*	7](#_Toc234324947)

[Figure S3: *Intention to Use the Strategies in the Future*	8](#_Toc234324948)

[Table S2: *Short-term Effects: Meal Type as Moderator*	9](#_Toc234324949)

[Table S3: *Long-term Effects: Meal Type as Moderator*	11](#_Toc234324950)

[Table S4: *Short-term Effects: Trait Self-control as Moderator*	13](#_Toc234324951)

[Table S5: *Long-term Effects: Trait Self-control as Moderator*	14](#_Toc234324952)

[Table S6: *Short-term Effects: Motivation as Moderator*	16](#_Toc234324953)

[Table S7: *Long-term Effects: Motivation as Moderator*	17](#_Toc234324954)

# Table S1

*Intervention Messages (translated)*

| Strategy | Example 1 | Example 2 | Example 3 | Example 4 |
| --- | --- | --- | --- | --- |
| Situation selection | | | | |
| approach | Seek out situations that make it easier for you to avoid meat.  Example: Choose restaurants that offer a wide range of appealing vegetarian dishes | Seek out situations that make it easier for you to avoid meat.  Example: Buy a sufficient amount of tasty vegetarian foods. | Seek out situations that make it easier for you to avoid meat.  Example: Make sure you have appealing vegetarian options available at home. | Seek out situations that make it easier for you to avoid meat.  Example: Plan meals in advance that are vegetarian. |
| avoidance | Avoid situations that make it harder for you to avoid meat.  Example: Avoid restaurants that specialize in meat and offer few or no vegetarian options. | Avoid situations that make it harder for you to avoid meat.  Example: Avoid social situations where meat is typically the main focus. | Avoid situations that make it harder for you to avoid meat.  Example: Avoid eating as a guest at places where meat dishes are usually prepared. | Avoid situations that make it harder for you to avoid meat.  For example: Avoid cooking and eating with people who pressure you to eat meat. |
| Situation modification | | | | |
| Modify the meal | Modify a meal in advance so that it is easier to eat less meat.  Example: Cook new vegetarian dishes or replace meat with vegetarian alternatives. | Modify a meal in advance so that it is easier to eat less meat.  Example: When you are at a restaurant, cafeteria, or canteen, order a dish with little or no meat. | Modify a meal in advance so that it is easier to eat less meat.  Example: Bring vegetarian food to difficult situations, e.g., to a barbecue party. | Modify a meal in advance so that it is easier to eat less meat.  Example: When cooking a meat dish, prepare only a small portion. |
| Modify social circumstances | Modify your social environment so that reducing meat consumption becomes easier.  Example: Try to encourage people you are eating with to order a vegetarian dish as well. | Modify your social environment so that reducing meat consumption becomes easier.  Example: Try to encourage people you cook with at home to eat less meat as well. | Modify your social environment so that reducing meat consumption becomes easier.  Example: Ask people you are dining with if they would like to share dishes so that meat may still be eaten, but less of it overall. | Modify your social environment so that reducing meat consumption becomes easier.  Example: Explain to people you eat with why you want to eat less meat. |
| Modify somatic environment/Reduce hunger | Reduce your hunger before challenging situations so that it is easier for you to avoid meat.  Example: Eat something vegetarian before going to a barbecue. | Reduce your hunger before challenging situations so that it is easier for you to avoid meat.  Example: Eat something vegetarian before meeting up with friends who typically want to eat meat. | Reduce your hunger before challenging situations so that it is easier for you to avoid meat.  Example: Eat something vegetarian before going to a special occasion where meat dishes are typically the main focus. | Reduce your hunger before challenging situations so that it is easier for you to avoid meat.  Example: Eat something vegetarian before you go shopping so that you don't shop while hungry. |
| Attentional Deployment | | | | |
| Approach | If you find yourself in a difficult situation, focus your attention on aspects that make it easier to reduce your meat consumption.  Example: Take a close look at the vegetarian dishes on the menu at the restaurant. | If you find yourself in a difficult situation, focus your attention on aspects that make it easier to reduce your meat consumption.  Example: When shopping, take a close look at the vegetarian foods. | If you find yourself in a difficult situation, focus your attention on aspects that make it easier to reduce your meat consumption.  Example: At a barbecue party, take a close look at the vegetarian foods (e.g., meat substitutes, salads). | If you find yourself in a difficult situation, focus your attention on aspects that make it easier to reduce your meat consumption.  Example: When eating with other people who eat meat, concentrate on your own plate. |
| Avoidance | If you find yourself in a difficult situation, shift your attention away from aspects that make it difficult to reduce your meat consumption.  Example: At a restaurant, ignore the meat dishes on the menu by quickly turning the page. | If you find yourself in a difficult situation, shift your attention away from aspects that make it difficult to reduce your meat consumption.  Example: When shopping, ignore foods containing meat by consciously looking away from them. | If you find yourself in a difficult situation, shift your attention away from aspects that make it difficult to reduce your meat consumption.  Example: At a barbecue, ignore the meat by deliberately looking away from it. | If you find yourself in a difficult situation, shift your attention away from aspects that make it difficult to reduce your meat consumption.  Example: When eating with other people, ignore the meat on their plates by consciously looking away from it. |
| Cognitive Change | | | | |
| Thinking of negative consequences | If you find yourself in a difficult situation, think about the negative consequences of meat consumption.  Example: Think about the animal suffering caused by animal husbandry and slaughter. | If you find yourself in a difficult situation, think about the negative consequences of meat consumption.  Example: Think about the high environmental impact of animal husbandry, including land and water use and greenhouse gas emissions such as CO2. | If you find yourself in a difficult situation, think about the negative consequences of meat consumption.  Example: Think about the negative health consequences of red and processed meat, such as an increased risk of cardiovascular disease. | If you find yourself in a difficult situation, think about the negative consequences of meat consumption.  Example: Remember that you may regret eating meat later. |
| Thinking of positive consequences | If you find yourself in a difficult situation, think about the negative consequences of eating meat.  Example: Think of the animals that are spared by not eating meat. | If you find yourself in a difficult situation, think about the negative consequences of eating meat.  Example: Think about the positive environmental impacts, including savings in land use, water consumption, and greenhouse gas emissions (such as CO2). | If you find yourself in a difficult situation, think about the negative consequences of eating meat.  Example: Think about the positive health effects, like a lower risk of heart disease. | If you find yourself in a difficult situation, think about the negative consequences of eating meat.  Example: Remember that you may be proud of yourself later for having given up meat. |
| Response modulation | If you find yourself in a difficult situation, use your willpower to resist eating meat.  Example: If you are in a restaurant, cafeteria, or canteen and delicious meat dishes are on offer, stay strong and suppress your craving for meat. | If you find yourself in a difficult situation, use your willpower to resist eating meat.  Example: If you are a guest at someone's house and they have prepared meat dishes, stay strong and suppress your desire for meat. | If you find yourself in a difficult situation, use your willpower to resist eating meat.  Example: If there are many delicious meat dishes available due to a special occasion, remain strong and suppress your desire for meat. | If you find yourself in a difficult situation, use your willpower to resist eating meat.  Example: When eating with people who typically eat meat, stay strong and suppress your desire for meat. |

*Note.* Situational strategies are shown on a white background, intrapsychic strategies on a grey background.

# Figure S1

*Perceived Strategy Effectiveness*

# Figure S2

*Perceived Strategy Feasibility*

# Figure S3

*Intention to Use the Strategies in the Future*

# Table S2

*Bayesian Multilevel Two-Part Model* *Predicting Meat Consumption from Baseline to Intervention Including Meal Type as a Moderator (Breakfast is the Reference Category)*

|  | Zero part | | | | Continuous part | | | | |
| --- | --- | --- | --- | --- | --- | --- | --- | --- | --- |
|  |  |  | 95% CI | | |  |  | 95% CI | |
|  | Estimate | *SE* | *LL* | *UL* | | Estimate | *SE* | *LL* | *UL* |
| *Fixed effects* |  |  |  |  | |  |  |  |  |
| Intercept | **1.38** | **0.10** | **1.19** | **1.56** | | **4.18** | **0.04** | **4.10** | **4.26** |
| Phase (0 = Baseline,  1 = Intervention) | **0.70** | **0.11** | **0.49** | **0.90** | | -0.07 | 0.05 | -0.16 | 0.02 |
| Strategies vs. Control | -0.09 | 0.21 | -0.51 | 0.31 | | 0.02 | 0.09 | -0.16 | 0.19 |
| Situational vs. Intrapsychic | -0.20 | 0.27 | -0.73 | 0.32 | | **0.30** | **0.12** | **0.07** | **0.55** |
| Combined vs. Single | -0.20 | 0.22 | -0.64 | 0.23 | | 0.00 | 0.10 | -0.18 | 0.19 |
| Strategies vs. Control *Phase | **0.48** | **0.23** | **0.04** | **0.93** | | -0.04 | 0.09 | -0.23 | 0.14 |
| Situational vs. Intrapsychic *Phase | 0.56 | 0.29 | -0.01 | 1.16 | | -0.15 | 0.13 | -0.41 | 0.10 |
| Combined vs. Single *Phase | **0.82** | **0.26** | **0.32** | **1.32** | | 0.02 | 0.11 | -0.18 | 0.23 |
| Lunch vs. Breakfast | **-1.14** | **0.10** | **-1.34** | **-0.94** | | **0.59** | **0.04** | **0.51** | **0.67** |
| Dinner vs. Breakfast | **-1.68** | **0.10** | **-1.89** | **-1.48** | | **0.64** | **0.04** | **0.56** | **0.71** |
| Lunch vs. Breakfast *Phase | -0.13 | 0.12 | -0.38 | 0.11 | | -0.08 | 0.05 | -0.18 | 0.02 |
| Dinner vs. Breakfast *Phase | -0.07 | 0.13 | -0.32 | 0.17 | | -0.04 | 0.05 | -0.14 | 0.05 |
| Lunch vs. Breakfast* Strategies vs. Control | 0.07 | 0.23 | -0.38 | 0.55 | | -0.16 | 0.09 | -0.34 | 0.01 |
| Lunch vs. Breakfast* Situational vs. Intrapsychic | 0.39 | 0.29 | -0.18 | 0.97 | | **-0.25** | **0.12** | **-0.48** | **-0.01** |
| Lunch vs. Breakfast* Combined vs. Single | -0.01 | 0.24 | -0.48 | 0.46 | | -0.03 | 0.09 | -0.22 | 0.16 |
| Dinner vs. Breakfast* Strategies vs. Control | 0.00 | 0.23 | -0.45 | 0.47 | | -0.13 | 0.09 | -0.29 | 0.05 |
| Dinner vs. Breakfast* Situational vs. Intrapsychic | **0.80** | **0.30** | **0.23** | **1.39** | | -0.19 | 0.11 | -0.41 | 0.03 |
| Dinner vs. Breakfast* Combined vs. Single | -0.23 | 0.25 | -0.24 | 0.70 | | -0.06 | 0.09 | -0.24 | 0.11 |
| Lunch vs. Breakfast* Strategies vs. Control  *Phase | -0.26 | 0.28 | -0.82 | 0.30 | | 0.18 | 0.11 | -0.03 | 0.40 |
| Lunch vs. Breakfast* Situational vs. Intrapsychic  *Phase | -0.41 | 0.35 | -1.11 | 0.27 | | 0.06 | 0.15 | -0.22 | 0.35 |
| Lunch vs. Breakfast* Combined vs. Single  *Phase | -0.52 | 0.31 | -1.13 | 0.09 | | 0.01 | 0.12 | -0.23 | 0.24 |
| Dinner vs. Breakfast* Strategies vs. Control  *Phase | -0.29 | 0.28 | -0.84 | 0.25 | | 0.07 | 0.10 | -0.14 | 0.27 |
| Dinner vs. Breakfast* Situational vs. Intrapsychic  *Phase | **-0.75** | **0.36** | **-1.45** | **-0.05** | | 0.12 | 0.14 | -0.15 | 0.39 |
| Dinner vs. Breakfast* Combined vs. Single  *Phase | **-0.65** | **0.31** | **-1.25** | **-0.05** | | 0.04 | 0.12 | -0.19 | 0.26 |
| *Random effects* |  |  |  |  | |  |  |  |  |
| SD Intercept | **0.64** | **0.06** | **0.52** | **0.76** | | **0.30** | **0.02** | **0.26** | **0.34** |
| SD Phase | **0.38** | **0.09** | **0.19** | **0.54** | | **0.16** | **0.03** | **0.11** | **0.21** |

*Note*. CI = credible interval, *LL* = lower limit, *UL* = upper limit. The zero part predicts the log-odds of a meat-free meal. The continuous part predicts the log amount of meat eaten in grams if a meal included meat. Credible intervals that do not include zero are presented in bold. *N* = 11946 observations (meals) across 156 participants.

**Interpretation of Table S2**

At baseline, the odds of consuming a meat-free meal were, on average, 68% lower at lunch than at breakfast (exp(−1.14) = 0.32) and 81% lower at dinner than at breakfast (exp(-1.68) = 0.19). Among meals containing meat, the amount of meat consumed was, on average, 80% higher at lunch than at breakfast (exp(0.59) = 1.80) and 90% higher at dinner than at breakfast (exp(0.64) = 1.90). The three-way interactions indicate that the relative short-term intervention effects of situational (vs. intrapsychic) strategies and combined (vs. single) strategies on choosing meat-free meals were stronger for breakfast than for dinner. No other three-way interactions showed credible effects. However, these analyses were exploratory, and the model is highly complex and likely underpowered to detect three-way interactions. Accordingly, these findings should be interpreted with caution and require replication in future research.

# Table S3

*Bayesian Multilevel Two-Part Model* *Predicting Meat Consumption from Baseline to Follow-up Including Meal Type as a Moderator (Breakfast is the Reference Category)*

|  | Zero part | | | | Continuous part | | | | |
| --- | --- | --- | --- | --- | --- | --- | --- | --- | --- |
|  |  |  | 95% CI | | |  |  | 95% CI | |
|  | Estimate | *SE* | *LL* | *UL* | | Estimate | *SE* | *LL* | *UL* |
| *Fixed effects* |  |  |  |  | |  |  |  |  |
| Intercept | **1.38** | **0.10** | **1.19** | **1.56** | | 4.19 | **0.04** | **4.10** | **4.27** |
| Phase (0 = Baseline,  1 = Follow-up) | **0.99** | **0.17** | **0.66** | **1.32** | | 0.05 | 0.07 | -0.19 | 0.09 |
| Strategies vs. Control | -0.22 | 0.23 | -0.66 | 0.21 | | 0.01 | 0.10 | -0.19 | 0.21 |
| Situational vs. Intrapsychic | -0.03 | 0.29 | -0.61 | 0.55 | | 0.25 | 0.14 | -0.01 | 0.51 |
| Combined vs. Single | 0.12 | 0.24 | -0.60 | 0.35 | | 0.00 | 0.11 | -0.21 | 0.21 |
| Strategies vs. Control *Phase | 0.63 | 0.35 | -0.06 | 1.33 | | 0.08 | 0.15 | -0.21 | 0.37 |
| Situational vs. Intrapsychic *Phase | **0.97** | **0.47** | **0.04** | **1.91** | | -0.25 | 0.20 | -0.63 | 0.14 |
| Combined vs. Single *Phase | **0.92** | **0.43** | **1.78** | **0.07** | | -0.13 | 0.18 | -0.48 | 0.23 |
| Lunch vs. Breakfast | **-1.06** | **0.11** | **-1.28** | **-0.86** | | **0.57** | **0.04** | **0.48** | **0.65** |
| Dinner vs. Breakfast | **-1.71** | **0.11** | **-1.93** | **-1.50** | | **0.65** | **0.04** | **0.56** | **0.73** |
| Lunch vs. Breakfast *Phase | **-0.37** | **0.18** | **-0.73** | **-0.02** | | **-0.21** | **0.08** | **-0.36** | **-0.06** |
| Dinner vs. Breakfast *Phase | -0.18 | 0.18 | -0.55 | 0.17 | | **-0.19** | **0.07** | **-0.33** | **-0.05** |
| Lunch vs. Breakfast* Strategies vs. Control | 0.11 | 0.25 | -0.37 | 0.62 | | -0.17 | 0.10 | -0.35 | 0.02 |
| Lunch vs. Breakfast* Situational vs. Intrapsychic | 0.10 | 0.31 | -0.52 | 0.72 | | -0.21 | 0.13 | -0.47 | 0.04 |
| Lunch vs. Breakfast* Combined vs. Single | 0.00 | 0.26 | -0.50 | 0.53 | | 0.00 | 0.10 | -0.20 | 0.20 |
| Dinner vs. Breakfast* Strategies vs. Control | 0.08 | 0.25 | -0.41 | 0.56 | | -0.11 | 0.09 | -0.29 | 0.08 |
| Dinner vs. Breakfast* Situational vs. Intrapsychic | 0.56 | 0.32 | -0.06 | 1.18 | | -0.15 | 0.12 | -0.38 | 0.09 |
| Dinner vs. Breakfast* Combined vs. Single | -0.33 | 0.26 | -0.17 | 0.85 | | -0.02 | 0.10 | -0.21 | 0.16 |
| Lunch vs. Breakfast* Strategies vs. Control  *Phase | -0.48 | 0.39 | -1.23 | 0.28 | | 0.05 | 0.16 | -0.26 | 0.36 |
| Lunch vs. Breakfast* Situational vs. Intrapsychic  *Phase | -0.79 | 0.52 | -1.77 | 0.27 | | 0.21 | 0.22 | -0.22 | 0.65 |
| Lunch vs. Breakfast* Combined vs. Single  *Phase | -0.28 | 0.47 | -1.21 | 0.58 | | 0.20 | 0.20 | -0.19 | 0.58 |
| Dinner vs. Breakfast* Strategies vs. Control  *Phase | -0.62 | 0.39 | -1.39 | 0.12 | | -0.05 | 0.16 | -0.35 | 0.26 |
| Dinner vs. Breakfast* Situational vs. Intrapsychic  *Phase | **-1.19** | **0.51** | **-2.21** | **-0.17** | | 0.12 | 0.21 | -0.30 | 0.52 |
| Dinner vs. Breakfast* Combined vs. Single  *Phase | -0.78 | 0.46 | -1.69 | 0.09 | | 0.22 | 0.19 | -0.59 | 0.15 |
| *Random effects* |  |  |  |  | |  |  |  |  |
| SD Intercept | **0.64** | **0.07** | **0.51** | **0.78** | | **0.31** | **0.03** | **0.27** | **0.36** |
| SD Phase | **0.89** | **0.11** | **0.68** | **1.12** | | **0.27** | **0.04** | **0.20** | **0.35** |

*Note*. CI = credible interval, *LL* = lower limit, *UL* = upper limit. The zero part predicts the log-odds of a meat-free meal. The continuous part predicts the log amount of meat eaten in grams if a meal included meat. Credible intervals that do not include zero are presented in bold. *N* = 5235 observations (meals) across 132 participants.

**Interpretation of Table S3**

At baseline, the odds of consuming a meat-free meal were, on average, 65% lower at lunch than at breakfast (exp(−1.06) = 0.35) and 82% lower at dinner than at breakfast (exp(-1.71) = 0.18). Among meals containing meat, the amount of meat consumed was, on average, 77% higher at lunch than at breakfast (exp(0.57) = 1.77) and 92% higher at dinner than at breakfast (exp(0.65) = 1.92). The three-way interactions indicate that the relative long-term intervention effect of situational (vs. intrapsychic) strategies on choosing meat-free meals was stronger for breakfast than for dinner. No other three-way interactions showed credible effects. However, these analyses were exploratory, and the model is highly complex and likely underpowered to detect three-way interactions. Accordingly, these findings should be interpreted with caution and require replication in future research.

# Table S4

*Bayesian Multilevel Two-Part Model* *Predicting Meat Consumption from Baseline to Intervention*

*Including Trait Self-Control (TSC) as a Moderator*

|  | Zero part | | | | Continuous part | | | | |
| --- | --- | --- | --- | --- | --- | --- | --- | --- | --- |
|  |  |  | 95% CI | | |  |  | 95% CI | |
|  | Estimate | *SE* | *LL* | *UL* | | Estimate | *SE* | *LL* | *UL* |
| *Fixed effects* |  |  |  |  | |  |  |  |  |
| Intercept | **0.38** | **0.06** | **0.27** | **0.50** | | **4.72** | **0.03** | **4.66** | **4.78** |
| Phase (0 = Baseline,  1 = Intervention) | **0.56** | **0.05** | **0.46** | **0.66** | | **-0.12** | **0.02** | **-0.16** | **-0.08** |
| Strategies vs. Control | -0.05 | 0.13 | -0.32 | 0.21 | | -0.10 | 0.06 | -0.23 | 0.02 |
| Situational vs. Intrapsychic | 0.22 | 0.17 | -0.12 | 0.57 | | 0.10 | 0.08 | -0.06 | 0.26 |
| Combined vs. Single | -0.11 | 0.14 | -0.38 | 0.16 | | -0.06 | 0.07 | -0.19 | 0.08 |
| Strategies vs. Control *Phase | 0.24 | 0.12 | 0.00 | 0.47 | | 0.07 | 0.05 | -0.02 | 0.16 |
| Situational vs. Intrapsychic *Phase | 0.13 | 0.15 | -0.15 | 0.42 | | -0.05 | 0.06 | -0.17 | 0.07 |
| Combined vs. Single *Phase | **0.32** | **0.13** | **0.07** | **0.57** | | 0.07 | 0.05 | -0.03 | 0.16 |
| TSC | -0.08 | 0.10 | -0.27 | 0.12 | | **-0.14** | **0.05** | **-0.23** | **-0.04** |
| TSC*Phase | -0.03 | 0.09 | -0.20 | 0.15 | | 0.05 | 0.03 | -0.02 | 0.12 |
| TSC*Strategies vs.  Control | 0.09 | 0.23 | -0.36 | 0.54 | | -0.04 | 0.11 | -0.25 | 0.18 |
| TSC*Situational vs. Intrapsychic | -0.05 | 0.28 | -0.61 | 0.49 | | -0.05 | 0.14 | -0.32 | 0.22 |
| TSC*Combined vs.  Single | 0.10 | 0.25 | -0.38 | 0.60 | | 0.07 | 0.12 | -0.16 | 0.31 |
| TSC*Strategies vs. Control*Phase | -0.03 | 0.21 | -0.42 | 0.36 | | -0.04 | 0.08 | -0.20 | 0.11 |
| TSC*Situational vs. Intrapsychic*Phase | -0.06 | 0.25 | -0.57 | 0.42 | | -0.03 | 0.10 | -0.22 | 0.17 |
| TSC*Combined vs. Single*Phase | -0.03 | 0.22 | -0.40 | 0.46 | | -0.04 | 0.09 | -0.21 | 0.13 |
| *Random effects* |  |  |  |  | |  |  |  |  |
| SD Intercept | **0.56** | **0.06** | **0.45** | **0.69** | | **0.31** | **0.02** | **0.26** | **0.36** |
| SD Phase | **0.31** | **0.08** | **0.14** | **0.46** | | **0.12** | **0.04** | **0.02** | **0.19** |

*Note*. CI = credible interval, *LL* = lower limit, *UL* = upper limit. The zero part predicts the log-odds of a meat-free meal. The continuous part predicts the log amount of meat eaten in grams if a meal included meat. Credible intervals that do not include zero are presented in bold. *N* = 11946 observations (meals) across 156 participants.

# Table S5

*Bayesian Multilevel Two-Part Model* *Predicting Meat Consumption from Baseline to Follow-up*

*Including Trait Self-Control (TSC) as a Moderator*

|  | Zero part | | | | Continuous part | | | | |
| --- | --- | --- | --- | --- | --- | --- | --- | --- | --- |
|  |  |  | 95% CI | | |  |  | 95% CI | |
|  | Estimate | *SE* | *LL* | *UL* | | Estimate | *SE* | *LL* | *UL* |
| *Fixed effects* |  |  |  |  | |  |  |  |  |
| Intercept | **0.36** | **0.06** | **0.23** | **0.48** | | **4.72** | **0.03** | **4.66** | **4.79** |
| Phase (0 = Baseline,  1 = Follow-up) | **0.70** | **0.10** | **0.51** | **0.88** | | **-0.11** | **0.04** | **-0.19** | **-0.04** |
| Strategies vs. Control | -0.14 | 0.14 | -0.42 | 0.15 | | -0.10 | 0.07 | -0.24 | 0.04 |
| Situational vs. Intrapsychic | 0.19 | 0.19 | -0.17 | 0.56 | | 0.08 | 0.09 | -0.10 | 0.26 |
| Combined vs. Single | 0.00 | 0.15 | -0.30 | 0.30 | | -0.03 | 0.08 | -0.18 | 0.12 |
| Strategies vs. Control*Phase | 0.16 | 0.21 | -0.26 | 0.58 | | 0.08 | 0.08 | -0.07 | 0.23 |
| Situational vs. Intrapsychic*Phase | 0.18 | 0.29 | -0.39 | 0.75 | | -0.08 | 0.10 | -0.28 | 0.11 |
| Combined vs. Single*Phase | **0.45** | **0.23** | **0.01** | **0.89** | | 0.09 | 0.09 | -0.09 | 0.26 |
| TSC | -0.05 | 0.11 | -0.27 | 0.16 | | **-0.19** | **0.05** | **-0.29** | **-0.09** |
| TSC*Phase | -0.09 | 0.16 | -0.40 | 0.22 | | 0.05 | 0.06 | -0.06 | 0.17 |
| TSC*Strategies vs. Control | 0.12 | 0.25 | -0.37 | 0.61 | | -0.10 | 0.12 | -0.33 | 0.13 |
| TSC*Situational vs. Intrapsychic | -0.16 | 0.32 | -0.81 | 0.45 | | -0.01 | 0.16 | -0.32 | 0.29 |
| TSC*Combined vs. Single | -0.02 | 0.27 | -0.55 | 0.50 | | 0.08 | 0.13 | -0.18 | 0.33 |
| TSC*Strategies vs. Control*Phase | -0.18 | 0.36 | -0.88 | 0.54 | | -0.07 | 0.13 | -0.32 | 0.20 |
| TSC*Situational vs. Intrapsychic*Phase | 0.01 | 0.47 | -0.90 | 0.92 | | 0.04 | 0.18 | -0.30 | 0.40 |
| TSC*Combined vs. Single*Phase | -0.41 | 0.40 | -1.18 | 0.38 | | 0.04 | 0.16 | -0.28 | 0.35 |
| *Random effects* |  |  |  |  | |  |  |  |  |
| SD Intercept | **0.57** | **0.06** | **0.45** | **0.70** | | **0.31** | **0.03** | **0.26** | **0.37** |
| SD Phase | **0.78** | **0.11** | **0.58** | **0.99** | | **0.25** | **0.04** | **0.17** | **0.33** |

*Note*. CI = credible interval, *LL* = lower limit, *UL* = upper limit. The zero part predicts the log-odds of a meat-free meal. The continuous part predicts the log amount of meat eaten in grams if a meal included meat. Credible intervals that do not include zero are presented in bold. *N* = 5235 observations (meals) across 132 participants.

**Interpretation of Tables S4 and S5**

Results show that at baseline higher trait self-control was associated with smaller meat portion sizes when meat was consumed. No credible moderation effects of trait self-control were observed in either the short-term or the long-term analyses. However, these analyses were exploratory, and the model is highly complex and likely underpowered to detect three-way interactions. Accordingly, these findings should be interpreted with caution and require replication in future research.

# Table S6

*Bayesian Multilevel Two-Part Model* *Predicting Meat Consumption from Baseline to Intervention*

*Including Motivation as a Moderator*

|  | Zero part | | | | Continuous part | | | | |
| --- | --- | --- | --- | --- | --- | --- | --- | --- | --- |
|  |  |  | 95% CI | | |  |  | 95% CI | |
|  | Estimate | *SE* | *LL* | *UL* | | Estimate | *SE* | *LL* | *UL* |
| *Fixed effects* |  |  |  |  | |  |  |  |  |
| Intercept | **0.40** | **0.06** | **0.29** | **0.52** | | **4.73** | **0.03** | **4.67** | **4.78** |
| Phase (0 = Baseline,  1 = Intervention) | **0.55** | **0.05** | **0.45** | **0.65** | | **-0.13** | **0.02** | **-0.17** | **-0.09** |
| Strategies vs. Control | -0.05 | 0.13 | -0.32 | 0.21 | | -0.10 | 0.07 | -0.23 | 0.03 |
| Situational vs. Intrapsychic | 0.21 | 0.16 | -0.10 | 0.54 | | 0.12 | 0.08 | -0.04 | 0.28 |
| Combined vs. Single | -0.03 | 0.14 | -0.31 | 0.25 | | -0.04 | 0.08 | -0.19 | 0.10 |
| Strategies vs. Control*Phase | **0.22** | **0.11** | **0.01** | **0.44** | | 0.06 | 0.05 | -0.03 | 0.15 |
| Situational vs. Intrapsychic*Phase | 0.14 | 0.14 | -0.13 | 0.41 | | -0.06 | 0.06 | -0.18 | 0.05 |
| Combined vs. Single*Phase | 0.23 | 0.12 | -0.01 | 0.47 | | 0.06 | 0.05 | -0.04 | 0.16 |
| Motivation | -0.03 | 0.06 | -0.13 | 0.08 | | -0.02 | 0.03 | -0.07 | 0.03 |
| Motivation*Phase | **0.16** | **0.05** | **0.07** | **0.25** | | -0.01 | 0.02 | -0.05 | 0.03 |
| Motivation*Strategies vs. Control | **-0.33** | **0.12** | **-0.57** | **-0.09** | | 0.03 | 0.06 | -0.09 | 0.14 |
| Motivation*Situational vs. Intrapsychic | -0.10 | 0.15 | -0.40 | 0.19 | | 0.06 | 0.08 | -0.09 | 0.21 |
| Motivation*Combined vs. Single | -0.21 | 0.15 | -0.50 | 0.08 | | -0.03 | 0.07 | -0.17 | 0.12 |
| Motivation*Strategies vs. Control*Phase | 0.15 | 0.10 | -0.05 | 0.34 | | -0.03 | 0.04 | -0.11 | 0.05 |
| Motivation*Situational vs. Intrapsychic*Phase | 0.03 | 0.13 | -0.22 | 0.29 | | -0.07 | 0.05 | -0.17 | 0.04 |
| Motivation*Combined vs. Single*Phase | 0.08 | 0.13 | -0.17 | 0.32 | | 0.09 | 0.05 | -0.01 | 0.19 |
| *Random effects* |  |  |  |  | |  |  |  |  |
| SD Intercept | **0.54** | **0.06** | **0.44** | **0.66** | | **0.31** | **0.02** | **0.27** | **0.36** |
| SD Phase | **0.24** | **0.09** | **0.07** | **0.41** | | **0.12** | **0.04** | **0.01** | **0.18** |

*Note*. CI = credible interval, *LL* = lower limit, *UL* = upper limit. The zero part predicts the log-odds of a meat-free meal. The continuous part predicts the log amount of meat eaten in grams if a meal included meat. Credible intervals that do not include zero are presented in bold. *N* = 11946 observations (meals) across 156 participants.

# Table S7

*Bayesian Multilevel Two-Part Model* *Predicting Meat Consumption from Baseline to Follow-up*

*Including Motivation as a Moderator*

|  | Zero part | | | | Continuous part | | | | |
| --- | --- | --- | --- | --- | --- | --- | --- | --- | --- |
|  |  |  | 95% CI | | |  |  | 95% CI | |
|  | Estimate | *SE* | *LL* | *UL* | | Estimate | *SE* | *LL* | *UL* |
| *Fixed effects* |  |  |  |  | |  |  |  |  |
| Intercept | **0.37** | **0.06** | **0.25** | **0.50** | | **4.72** | **0.03** | **4.66** | **4.79** |
| Phase (0 = Baseline,  1 = Follow-up) | **0.69** | **0.09** | **0.51** | **0.87** | | **-0.12** | **0.04** | **-0.19** | **-0.05** |
| Strategies vs. Control | -0.13 | 0.14 | -0.40 | 0.14 | | -0.11 | 0.07 | -0.25 | 0.04 |
| Situational vs. Intrapsychic | 0.18 | 0.19 | -0.20 | 0.54 | | 0.11 | 0.10 | -0.08 | 0.30 |
| Combined vs. Single | 0.05 | 0.15 | -0.25 | 0.35 | | -0.02 | 0.08 | -0.18 | 0.15 |
| Strategies vs. Control*Phase | 0.14 | 0.21 | -0.26 | 0.56 | | 0.08 | 0.07 | -0.07 | 0.23 |
| Situational vs. Intrapsychic*Phase | 0.21 | 0.28 | -0.34 | 0.75 | | -0.11 | 0.10 | -0.31 | 0.08 |
| Combined vs. Single*Phase | 0.37 | 0.23 | -0.08 | 0.83 | | 0.07 | 0.09 | -0.10 | 0.24 |
| Motivation | -0.03 | 0.06 | -0.15 | 0.09 | | 0.00 | 0.03 | -0.06 | 0.06 |
| Motivation*Phase | 0.14 | 0.09 | -0.04 | 0.32 | | -0.01 | 0.03 | -0.07 | 0.05 |
| Motivation*Strategies vs. Control | **-0.34** | **0.14** | **-0.61** | **-0.08** | | 0.05 | 0.07 | -0.10 | 0.18 |
| Motivation*Situational vs. Intrapsychic | -0.13 | 0.17 | -0.46 | 0.20 | | 0.08 | 0.09 | -0.09 | 0.25 |
| Motivation*Combined vs. Single | -0.16 | 0.16 | -0.48 | 0.14 | | 0.01 | 0.08 | -0.16 | 0.17 |
| Motivation*Strategies vs. Control*Phase | 0.20 | 0.20 | -0.19 | 0.58 | | 0.03 | 0.07 | -0.11 | 0.17 |
| Motivation*Situational vs. Intrapsychic*Phase | 0.29 | 0.24 | -0.19 | 0.77 | | -0.14 | 0.09 | -0.31 | 0.02 |
| Motivation*Combined vs. Single*Phase | 0.13 | 0.24 | -0.32 | 0.61 | | 0.17 | 0.09 | 0.00 | 0.33 |
| *Random effects* |  |  |  |  | |  |  |  |  |
| SD Intercept | **0.54** | **0.06** | **0.43** | **0.67** | | **0.33** | **0.03** | **0.28** | **0.39** |
| SD Phase | **0.75** | **0.11** | **0.55** | **0.96** | | **0.23** | **0.04** | **0.14** | **0.32** |

*Note*. CI = credible interval, *LL* = lower limit, *UL* = upper limit. The zero part predicts the log-odds of a meat-free meal. The continuous part predicts the log amount of meat eaten in grams if a meal included meat. Credible intervals that do not include zero are presented in bold. *N* = 5235 observations (meals) across 132 participants.

**Interpretation of Tables S6 and S7**

Results show that regarding the short-term analysis, higher motivation was associated with a stronger increase in the odds of meat-free meals. No credible three-way interactions with motivation were observed in either the short-term or the long-term analyses. However, these analyses were exploratory, and the model is highly complex and likely underpowered to detect three-way interactions. Accordingly, these findings should be interpreted with caution and require replication in future research.
